# The quest for an effective and safe personalized cell therapy using epigenetic tools

**DOI:** 10.1186/s13148-016-0283-5

**Published:** 2016-11-16

**Authors:** T. A. L. Brevini, G. Pennarossa, E. F. M. Manzoni, C. E. Gandolfi, A. Zenobi, F. Gandolfi

**Affiliations:** Laboratory of Biomedical Embryology, Unistem, Università degli Studi di Milano, Via Celoria 10, 20133 Milan, Italy

**Keywords:** Epigenetic conversion, Epigenetics, iPSCs, Molecular medicine, Personalized medicine, Regenerative medicine, Small molecules

## Abstract

In the presence of different environmental cues that are able to trigger specific responses, a given genotype has the ability to originate a variety of different phenotypes. This property is defined as plasticity and allows cell fate definition and tissue specialization. Fundamental epigenetic mechanisms drive these modifications in gene expression and include DNA methylation, histone modifications, chromatin remodeling, and microRNAs. Understanding these mechanisms can provide powerful tools to switch cell phenotype and implement cell therapy.

Environmentally influenced epigenetic changes have also been associated to many diseases such as cancer and neurodegenerative disorders, with patients that do not respond, or only poorly respond, to conventional therapy. It is clear that disorders based on an individual’s personal genomic/epigenomic profile can rarely be successfully treated with standard therapies due to genetic heterogeneity and epigenetic alterations and a personalized medicine approach is far more appropriate to manage these patients.

We here discuss the recent advances in small molecule approaches for personalized medicine, drug targeting, and generation of new cells for medical application. We also provide prospective views of the possibility to directly convert one cell type into another, in a safe and robust way, for cell-based clinical trials and regenerative medicine.

## Background

Epigenetics is at the center of modern biology and medicine, since it is currently considered a fundamental tool to understand embryo development and stem cell biology, as well as to explain the relationship among an individual’s genetic background, the environmental influences, aging, and disease susceptibility.

The most exciting idea is that epigenetics may provide new clues to intervene at the junction between the genome and the environment, modifying the effects of deleterious genes [1]. It would be also useful to develop new strategies for disease prevention and therapy and to master tissue reprogramming in regenerative medicine.

In particular, during the last years, great attention was given to epigenetics in order to prevent, diagnose, and treat different diseases. Indeed, it has been demonstrated that malignant transformations as well as several disorders, such as autism, bipolar disorder, familial hypertrophic cardiomyopathy, schizophrenia, and syndromes, namely Prader-Willi, Angelman, Beckwith-Wiedemann, and Silver-Russell, are directly or indirectly caused by epigenetic alterations in form of mutation of DNA methylation or incorrect histone modifications [2–5]. In particular, DNA methyltransferase (DNMT) inhibiting nucleoside analogs, non-nucleoside analogs, and histone deacetylase (HDAC) inhibitors have been proposed as potential anti-cancer drugs. In parallel, several researches are focusing on the development of direct disease treatments with small molecules, based on individual personal genomic profile and epigenetic characteristics of each patient, in order to improve outcomes.

Presently, a growing problem is also represented by degenerative diseases that, despite decades of research, still lack effective cures. Regenerative medicine has earned increased attention and represents an attractive option as a potentially novel approach for the treatment of neurodegenerative, cardiovascular and liver diseases, diabetes, spinal cord injury, and corneal degeneration. In this field, the use of small molecules in cell reprogramming technology has allowed for the development of protocols that avoid the use of retroviral and/or lentiviral vectors, and the insertion of transgenes for the generation of induced pluripotent cells (iPSC). However, although these cells may represent a promising stem cell source, the induction of a stable pluripotent state and the deriving cell instability severely limits their use in cell therapy.

In order to circumvent these limits, a new small-molecule-based method able to directly convert a terminally differentiated cell into a different cell type has been recently proposed. This new approach demonstrated that it is possible to dynamically interact with cell genotype and phenotype through the use of epigenetic modifiers [2–7].

We here discuss the recent advances in small molecule approaches for drug targeting, personalized medicine, and generation of new cells for medical application. We also provide prospective views on the possibility to directly convert one cell type into another, in a safe and reproducible way, in order to obtain cells that may find application in clinical trials and regenerative medicine.

## Review

### Molecular basis of epigenetics

The molecular basis of epigenetics is a complex phenomenon that determines activation or silencing of certain genes, without changing the DNA sequence.

There are several types of epigenetic mechanisms that play an essential role in the regulation of chromatin structure and gene expression, namely histone post-translational modifications, covalent modification of DNA, small (21- to 26-nt) non-coding RNAs (ncRNAs), and recombination of non-genic DNA.

These processes are driven by different proteins that are usually categorized based on their molecular nature. In particular, the enzymes involved in epigenetic control are classified as epigenetic writers, epigenetic erasers, and epigenetic readers (see Table 1).

**Table 1:** Mechanisms involved in epigenetic control and related epigenetic enzymes. References

Mechanism	Writer	Eraser	Reader
DNA methylation	DNA methyltransferases (DNMT1, DNMT3) [67, 68]	DNA demethylation enzymes (TET) [69]	Methyl-CpG binding domains (MECP2, MBD1, MBD2, and MBD4) [70]
Histone lysine acetylation	Histone acetyltransferases (GCN5/PCAF, MYST, P300/CBP, SRC/p160) [71]	Histone deacetylases (HDAC1, HDAC2, HDAC3, HDAC4, HDAC5, HDAC6, HDAC7, HDAC8, HDAC9, HDAC10, HDAC11); Sir2-like proteins (SIRT1, SIRT2, SIRT3, SIRT4, SIRT5, SIRT6, SIRT7) [72]	Bromodomain, tandem PHD [73, 74]
Histone arginine methylation	Histone arginine methyltransferases (PRMT1, PRMT2, PRMT3, PRMT5, PRMT6, PRMT7, CARM1) [75]	Histone arginine demethylase (bifunctional arginine demethylase and lysyl-hydroxylase JMJD6) [76]	Tudor, ADD, WD40 [73, 74]
Histone lysine methylation	Histone lysine methyltransferases (EZH, SET1, SET2, SMYD, SUV39, SUV4-20, RIZ, SET8/PR-SET7, SET7/9, PRDM) [77]	Histone lysine demethylases Lysine-specific demethylase (LSD1, LSD2); Jumonji histone demethylases (JHDM1, JHDM2, JHDM3/JMJD2, JARID, JMJC, PHF2/PHF8, UTX/UTY) [77]	Chromodomain, ADD, ankyrin, BAH, chromobarrel Tudor, PHD fingers, MBT, ZF-CW, WD40, PWWP [73, 74]
Histone phosphorylation	Histone kinases (AGC, CaMK, CMGC, protein-tyrosine kinase, MEK) [78]	Histone phosphatases Serine/threonine phosphatases (PPP, PPM); tyrosine phosphatases (PTP, VH1-like dual-specificity phosphatase, cdc25) [79, 80]	Chromoshadow, 14.3.3 proteins, BIR, BRCT [73, 74]
Histone lysine ubiquitination	Histone lysine ubiquitinases (E1 enzyme, E2 ubiquitin conjugases); E3 ubiquitin-protein ligases (HECT domain, RING finger domain) [81]	Histone lysine deubiquitinases (UCH, USP, MJD, OTU, JAMM) [82]	Unknown
Histone arginine citrullination	Histone arginine deiminases (PAD1, PAD2, PAD3, PAD4, PAD6) [83]	Unknown	Unknown
Histone lysine biotinylation	Histone lysine biotinases (HLCS) [84]	Unknown	Unknown
Histone lysine ribosylation	Histone lysine ribosylases (PARP1) [85]	Histone lysine deribosylase (PARG) [85]	Unknown

The firsts catalyze modifications either on DNA, RNA, or histone proteins by adding of chemical groups on top of them. This group includes the following:

- Histone methyltransferases (HMTs), which are further subdivided into lysine methyltransferases (PKMTs) and arginine methyltransferases (PRMTs) according to their target residue
- Histone acetyltransferases (HATs)
- Enzymes that catalyze the phosphorylation of histone tails
- Ubiquitin-conjugating enzymes
- DNA methyltransferases (DNMTs)

In contrast, epigenetic erasers remove the structural modifications introduced by the writers. They comprise the following:

- Histone deacetylases (HDACs)
- Histone serine/threonine/tyrosine phosphatases
- Histone deubiquitinases (DUBs)
- Histone lysine/arginine demethylases
- DNA demethylation enzymes

Lastly, epigenetic readers are effector proteins that recognize specific structural units in nucleic acids and proteins and are recruited to specific marks on histones or nucleotides. Their structure is characterized by a cavity in which to accommodate a specific epigenetic mark. The interaction between the reader domain and the modified amino acid allows to distinguish similar epigenetic marks. Furthermore, they can be also contained in writer or eraser enzymes and are classified into four groups:

- Chromatin architectural proteins
- Chromatin remodeling enzymes
- Chromatin modifiers
- Adaptor proteins

### Epigenetic in medicine

During the last years, the understanding of genetic and epigenetic is becoming increasingly important for the prevention, diagnosis, and treatment of several diseases, and much attention has been given to molecular medicine. In this contest, it has been demonstrated that several disorders were directly or indirectly caused by epigenetic modifications in form of impaired DNA methylations or incorrect histone modifications [8]. Human diseases, such as autism, bipolar disorder, diabetes, familial hypertrophic cardiomyopathy, schizophrenia, and syndromes, namely Prader-Willi, Angelman, Beckwith-Wiedemann, and Silver-Russell, have been related to alteration of DNA methylation and modifications of normal imprinting patterns [9–11]. In particular, these human rare syndromes appear to be directly linked to aberrant expression of long ncRNAs [12]. They are involved in the epigenetic controls of coding genes, through the up- or down-regulation of messenger RNAs (mRNAs), methylation, and transcription of specific gene polymorphisms [13], thus exerting a powerful effect on a number of physiological processes. Their aberrant levels are likely to cause disorders associated with protein dysregulations [14]. Despite the present advances, regulatory mechanisms and functions of long non-coding RNA (lncRNA), and their association with the majority of the diseases, need to be further elucidated in order to improve patient management, as well as the prevention and treatment of the related genetic diseases.

It is well known that genetic aberrations can also promote malignant transformations. Many studies demonstrated that initiation and progression of several form of cancer are related to epigenetic aberrations that alter the complex functional interaction and balance between oncogenes and tumor suppressor genes [15, 16]. One of the main actors is hypermethylation of many tumor suppressor genes, such as those involved in DNA repair (BRCA1, MGMT and MLH1), signal transduction (RASSF1A), cell cycle regulation (p16INK4a), apoptosis (DAPK and TMS1), and angiogenesis (THBS1) [17–19]. Indeed, epigenetic disruption was one of the main abnormality identified in cancer cells [20] and might lead to gene activation, promoting overexpression of oncogenes, and might represents a fundamental mechanism of cancer development [17].

Alteration of normal patterns of covalent histone modifications is yet another hallmark of cancer. The most characteristic examples are, in this respect, related to the overexpression, mutations, and/or chromosomal translocations of histone acetylation/deacetylation (HAT/HDAC) and methylation/demethylation (HMT/HDM or sirtuins) enzymes [21].

In this context, the development of molecular medicine, the fast progress of the new epigenetic approaches, and the reversible nature of the epigenome offer great advances in the fields of drug targeting and personalized medicine.

Based on these observations, DNMT-inhibiting cytosine nucleoside analogs and non-nucleoside analogs (see Table 2) have been proposed as potential anti-cancer drugs. The most characterized nucleoside analogs, 5-azacytidine (Vidaza®) and 5-aza-2′-deoxycytidine or decitabine (Dacogen®), have been approved by the US Food and Drug Administration (FDA) and European Medicines Agency (EMA) for the treatment of myelodysplastic syndrome (MDS) and chronic myelomonocytic leukemia (CMML). Several clinical studies have also shown promising results in patients with acute myeloid leukemia (AML) and acute lymphoblastic leukemia (ALL) [22]. Preliminary experiments also demonstrated that dihydro-5-azacytidine (DHAC) and zebularine are less cytotoxic than the 5-aza-nucleosides in cultured cells and that are able to inhibit tumorigenesis in various cancer cell lines [23–27]. However, further studies are needed in order to demonstrate safety and efficacy and, eventually, enter into the clinical phase.

**Table 2:** List of nucleoside and non-nucleoside analog DNMT inhibitors

Nucleoside analogs	Non-nucleoside analogs
5-6-Dihydro-azacytidine	(−)-Epigallocatechin-3-galate
5-Fluoro-2-deoxycytidine	Curcumin
Azacytidine	Mithramycin A
CP-4200	Nanomycin A
Decitabine	Natural compounds: flavonoids
NPEOC-DAC	NSC-106084
SGI-110	NSC-14778
Zebularine	PRIMA-1
	Psammaplin A
	RG-108
	SGI-1027
	Synthetic compounds: procaine

Non-nucleoside analogs are also being studied. For instance, procainamide and its analog procaine have shown DNMT inhibitory effects in various cancer types, as well as other synthetic compounds, such as RG108, MG98, PRIMA-1, and SGI-1027, and natural compounds, namely flavonoids, psammaplin A, and curcumin. However, none of them have entered clinical development yet, since there is still a long way to go before we may obtain the identification of novel, selective, non-nucleoside DNMT inhibitors.

As described above, cancer cells can also be characterized by alterations of histone methyltransferases/demethylases and overexpression of histone deacetylases (HDACs). Several reports indicate that HDAC inhibitors are able to induce a cell cycle arrest at G1 or G2-M stage, cancer cell differentiation and apoptosis. Furthermore, these molecules can inhibit angiogenesis and metastasis and enhance cell sensitivity to chemotherapy [28]. Several HDACi are being tested in phase II–III trials as reported in Table 3 and include both natural and synthetic compounds [29]. Vorinostat and romidepsin are the first agents approved by the FDA and EMA for the treatment of progressive or recurrent cutaneous T cell lymphoma (CTCL) [30], while several other molecules, listed in Table 2, are in the early phases of clinical development [31].

**Table 3:** List of HDACs and their current status in clinical trials

Group	Example	Current status
Short-chain fatty acid	Valproic acid	Phase II CT
	Phenyl butyrate	Phase II CT
	Pivanex	Phase II CT
Hydroxamic acids	Vorinostat	FDA approved
	Panobinostat	Phase III CT
	Belinostat	Phase II CT
	Abexinostat	Phase II CT
	Resminostat	Phase II CT
	Givinostat	Phase II CT
	Dacinostat	Phase II CT
	Pracinostat	Phase II CT
Cyclic tetrapeptide	Romidepsin	FDA approved
	Apicidin	Phase II CT
	Trapoxin A	No data
Benzamide	Mocetinostat	Phase II CT
	Entinostat	Phase II CT
	Rocilinostat	Phase II CT

Currently, several clinical trials are testing the use of a different combination of DNMT and HDAC inhibitors, together with cyclin-dependent kinase inhibitors (CDKi) or proteasome inhibitors or engineered transcriptional factors [32–36].

Another new concept derives from the observation that patients with the same disease may have different symptoms and may not or only poorly respond to conventional therapy. This brings about the concept of “personalized medicine,” also known as “precision medicine.” This new branch of medicine, basically, encompasses the tailoring of medical treatment on the basis of individual characteristics, needs, and preferences of each patient, in order to improve outcomes [37]. As very prematurely stated by Hippocrates “It’s far more important to know what person the disease has than what disease the person has,” introducing for the first time the idea of the “individuality” of disease and the importance of prescription of “different” medicines to “different” patients.

In this contest, gene-expression profiling and genomic studies represent potential tools for improving patient management through their classification into clinically relevant subtypes for prevision therapy [38]. Although FDA has already approved some expression profiling platforms for clinical use, strong claims cannot yet be made about the clinical value of these signatures. Other important technological platforms are being developed to analyze epigenetic changes in DNA, microRNAs, and proteins. These allow to identify biomarkers for individual’s classification into subpopulations that differ in their susceptibility to a particular disease or in their response to a specific treatment. Furthermore, it is important to highlight that, although a few drugs used for personalized medicine have been approved by FDA, various challenges still exist, given the observation that each patient is unique and, similarly, displays a unique epigenomic signature.

### Epigenetic in stem cell research

Access to unlimited numbers of specific cell types represents the major goal in regenerative medicine. Recent advances in the stem cell field led to the production of iPSCs that were generated in 2006 through genetic reprogramming of adult somatic cells. Following these studies, several researchers succeeded in producing iPSCs. However, although various methodologies have been established for their derivation, the efficiency of iPSC induction remains low. Furthermore, the integration of transgenes severely limits their use in clinical studies [39]. Therefore, several reprogramming technologies that increase efficiency, accelerate kinetics, and eliminate the use of virus-mediated gene have been developed. Different approaches have been tested, from virus-free [40–42] to removable PiggyBac transposons [43], minicircle systems [44], and episomal systems [45]. Nevertheless, evidence persists demonstrating the problems related to residual exogenous DNA and chromosomal disruptions that result in harmful genetic alterations [46].

In order to circumvent these limits related to low efficiency and the introduction of exogenous transcription factors, small-molecule compounds have been used to modulate the epigenetic state increasing reprogramming efficiency, by inhibiting and activating, in reversible way, specific signaling pathways [47–50].

Huangfu et al. reported that the HDAC inhibitor, valproic acid (VPA), not only improves reprogramming efficiency by more than 100-fold but also enables efficient induction of human and murine iPSCs, without introduction of the myelocytomatosis oncogene (c-Myc) [47].

Subsequently, it was demonstrated that murine embryonic and adult fibroblasts could be reprogrammed by stimulating cells with a specific chemical combination of VPA, CHIR99021, 616452, and tranylcypromine (TCP), in the presence of a single transcription factor, POU domain, class 5, transcription factor 1 (*Oct-4*), without the use of transgenes for SRY (sex-determining region Y)-box 2 (*Sox2*), Kruppel-like factor 4 (gut) (*Klf4*), and *c-Myc* [48].

A recent study also reveals that endogenous pluripotency program can be re-activated through the use of small molecules that modulate molecular pathways nonspecifically related to pluripotency, without the introduction of exogenous genes. In that report, Hou et al. generated iPSCs from murine somatic cells at a frequency up to 0.2%, using a combination of seven small-molecule compounds, namely VPA, CHIR99021, 616452, TCP, forskolin (FSK), 2-methyl-5-hydroxytryptamine (2-Me-5HT), and D4476 [50]. In line with these findings, Moschidou et al. demonstrated that the use of VPA in combination with a low growth factor medium (embryonic stem cell (ESC) medium) is able to revert 82% of amniotic fluid cells into a pluripotent state that shares transcriptome identity with ESC and ability to form embryoid bodies (EB) and teratomas, as well as to differentiate into cell lineages deriving from all the three germ layers [51]. Similarly, endogenous pluripotency transcription factor genes were re-activated in adult human dermal fibroblasts using VPA, in the absence of any transgenes [52].

Although the exact mechanisms underlying iPSC generation still remain to be elucidated, these results suggest that epigenetic modifiers improve cell reprogramming altering chromatin structure and directly modulating the epigenetic enzymes. These events possibly drive cells to a more permissive state that allow changes in the epigenome, activating specific signaling pathways that influence cell fate during reprogramming processes.

Altogether, the data obtained represent a significant progress in cell reprogramming technology, with new approaches that avoid the use of retroviral and/or lentiviral vectors and the insertion of transgenes.

### Epigenetic and direct cell conversion: a new alternative

**Fig. 1:**
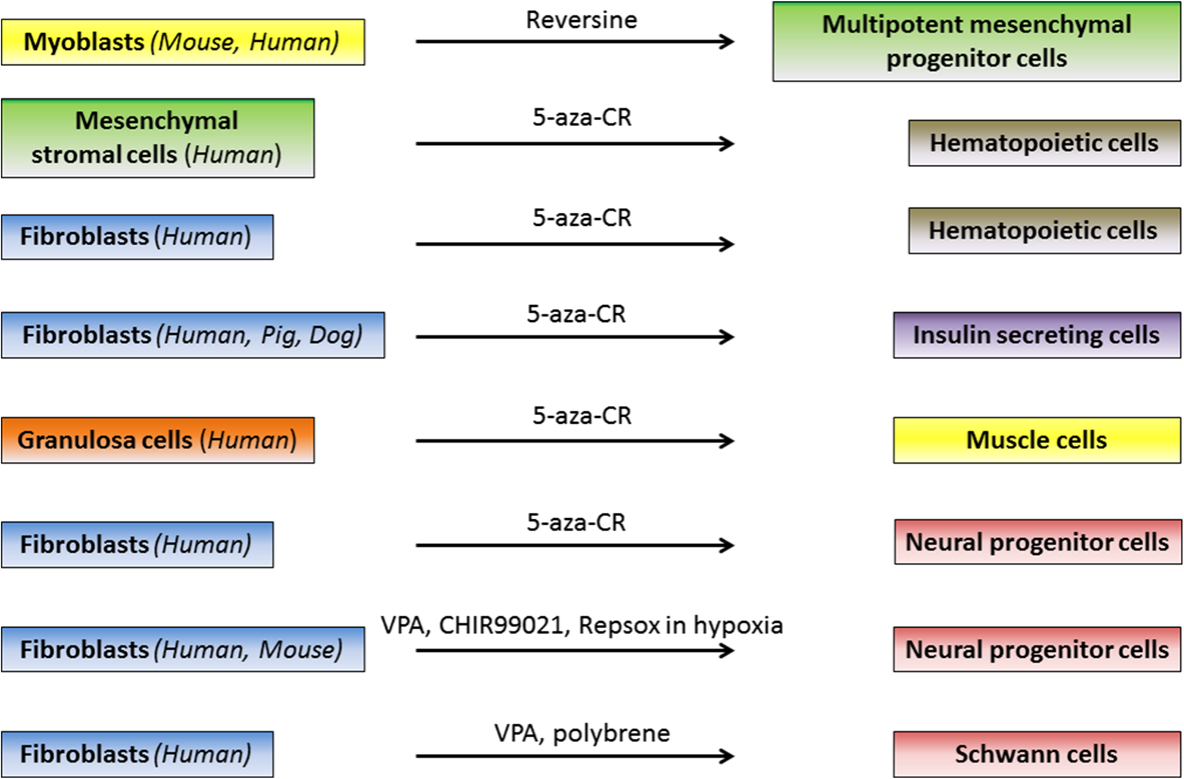
Schematic representation of epigenetic conversion experiments

In recent years, several protocols that avoid the use of virally or non-virally introduced exogenous factors as well as the establishment of a stable pluripotent state have been developed. These new approaches involve the use of small molecules and epigenetic modifiers in order to directly convert an adult mature cell into another differentiated cell type (Fig. 1).

The first paper reporting the ability of a small molecule to induce a de-differentiation in murine C2C12 myoblasts was published in 2004 [53]. In these experiments, cells were initially treated with a library of 50,000 small molecules for 4 days, with the final goal of identifying target compounds that can induce de-differentiation. The results obtained demonstrated that reversine, a 2,6-disubstituted purine, was able to increase cell plasticity, inducing lineage-committed myoblasts to become multipotent mesenchymal progenitor cells. The activity of this molecule was subsequently tested in several type of cells, including 3T3E1 osteoblasts [54], human primary skeletal myoblasts [54], and murine and human dermal fibroblasts [55], confirming the induction of an increased plasticity in treated cells.

More recent experiments demonstrated that a brief exposure to a demethylating agent can push cells to a less committed state, increasing their plasticity for a short window of time sufficient to re-address cells towards a different cell type [2–7]. The starting hypothesis was that the processes associated with differentiation are driven by several mechanisms. Among these, DNA methylation plays a fundamental role during both early embryonic development and cell lineage specification, causing silencing of large fraction of the genome and subsequent expression of gene essential for the maintenance of the differentiated and tissue-specific phenotype. Based on this, 5-azacytidine (5-aza-CR), a well-characterized DNMT inhibitor, was selected in order to remove the epigenetic “blocks” that are responsible for tissue specification [3–5, 7]. This drug is a chemical analog of cytosine, it can be incorporated into DNA and RNA, causing an increased effect in resting as well as in dividing cells, and it is known to be a direct inhibitor of methylation in newly synthesized DNA by blocking DNMT function [56]. These features give 5-aza-CR the ability to induce DNA hypomethylation, modify gene expression, and reactivate the transcription of silent genes in eukaryotic cells [57–62].

In agreement with these findings, human mesenchymal stromal cells (MSC) and skin fibroblasts were transformed into hematopoietic cells after an incubation with 5-aza-CR, granulocyte-macrophage colony-stimulating factor (GM-CSF), and stem cell factor (SCF) [2].

Moreover, our laboratory demonstrated that adult skin fibroblasts and granulosa cells, derived from different species, namely human [3, 5], porcine [4], and dog [63], can be converted into a different cell type, belonging to the same embryonic layer or even to a different one.

The “highly permissive state” enriched by cells, after 5-aza-CR exposure, was paralleled by decrease in global DNA methylation and was accompanied by significant changes in cell phenotype and a specific and consistent gene regulatory response. Indeed, after demethylating agent treatment, both cell types used in the experiments, adult skin fibroblasts and granulosa cells, exhibited reduced dimensions, increased nuclear volume, and highly de-condensed chromatin [3–5]. These observations are in agreement with the morphological features distinctive of highly plastic cells that contain more loosely packed chromatin than their differentiated counterparts, in order to maintain genes in a potentially open state and prepare them for future expression [64]. Preliminary data obtained in our laboratory with next-generation sequencing analysis of 5-aza-CR exposed cells indicate changes of several pathways, mostly related to histone transcription and cell adhesion. This suggests the possibility that, beside the well-known effect on DNMTs and cell methylation, 5-aza-CR action on cell plasticity and differentiation may take place through alternative mechanisms that require the involvement of novel cellular targets (manuscript under revision). Notably, this process is completely reversible and does not show toxic effects, since cells returned to their standard culture medium, reverted to their original phenotype within a few days. The absence of genotoxic effects is further supported by cytogenetic analysis showing that 5-aza-CR-treated cells maintained a normal karyotype throughout the entire length of the experiments [3–5]. We also demonstrated that, once cells entered into the higher plasticity window, they could easily be directed towards a different phenotype if they were exposed to specific differentiation stimuli.

In particular, skin fibroblasts of human, porcine, and canine origin were converted towards the pancreatic lineage, using a three-step induction protocol. This allowed cells to transit from the early endodermic and pancreatic differentiation stage to mature endocrine cells. At the end of the epigenetic conversion, cells formed large three-dimensional spherical structures, reminiscent of in vitro-cultured pancreatic islets. They expressed the main hormones and glucose sensor genes specific of pancreatic tissue and were able to actively release of C-peptide and insulin after exposure to 20 mM glucose, showing a dynamic response similar to pancreatic β cells, in which changes in ambient glucose represent the primary and physiological stimulus for insulin secretion. Furthermore, cell functionality was also demonstrated in vivo using immunodeficient severe combined immunodeficiency (SCID) mice whose β cells had been selectively destroyed with streptozotocin, demonstrating converted cell ability to restore normo-glycaemia and stably maintain mice glucose levels [3, 4].

The possibility to apply epigenetic conversion to different cell types has been demonstrated using granulosa cells as starting cell population and converting them into muscle cells through the use of 5-aza-CR followed by a 15-day culture with human recombinant vascular endothelial growth factor (VEGF) [5]. At the end of the conversion, over 80% of granulosa cells change the original phenotype and become elongated and multinucleated. These morphological changes were paralleled by the up-regulation of muscle-specific genes, such as desmin (DES), myosin heavy chain (MHC), and myogenic differentiation (MYOD). In contrast, markers distinctive of granulosa cells (cytokeratin 17 (KRT17), hyaluronan synthase 2 (HAS2), gremlin 1 (GREM1), and pentraxin 3 (PTX3)) were turned down.

In agreement with our results, the demethylating agent 5-aza-CR was also demonstrated to convert human foreskin fibroblasts into neural progenitor-like cells [6]. At the end of the 14-day neural conversion, cells down-regulated fibroblast specific protein 1 (FSP1) and expressed high levels of neural progenitor markers, namely SOX2, NESTIN, PAX6, EN1, LMX1A, and WNT1. Molecular switch was accompanied by morphological changes, with cells becoming smaller, acquiring radial arrangement, and producing neurosphere-like aggregates.

Cheng et al. reported that it is possible to convert human and murine fibroblasts into proliferating chemical-induced neural progenitor cells (ciNPC), using a cocktail containing inhibitors of histone deacetylation, glycogen synthase kinase, and TGF-β pathway under physiological hypoxic conditions (5% O_2_) [65].

Furthermore, recent experiments described the possibility to epigenetically convert human skin fibroblasts into mature Schwann cells through the use of the HDAC inhibitor VPA [66]. In that work, cells were stimulated with a two-step neural induction protocol, in order to obtain a transient population of proliferating neural precursors and, subsequently, terminally differentiated Schwann cells (iSCs), that showed neuro-supportive and myelination capacity, and expressed proteins specific of the peripheral nervous system.

## Conclusions

Altogether, the results accumulated during the last years have paved the way to the use of small molecules for personalized medicine, drug targeting, and the induction of changes in cell fate. Some of these molecules have been already approved for patient’s treatment and are currently used for the cure of disease caused be epigenetic aberrations, while other chemical compounds are tested in several clinical trials. In this context, various challenges still exist given the observation that each patient is unique and displays a unique epigenomic signature, and more studies are indeed in order to develop epigenetic biomarkers, technologies, and tools to classify individuals into subpopulations that differ in their susceptibility to a particular disease or in their response to a specific treatment.

Epigenetic modifiers are also been used to replace TFs for iPSC generation. Indeed, mouse and human iPSCs have been generated using a small-molecule-based reprogramming protocol, without the use of genetic material. However, although these cells may represent a promising stem cell source, it is important to highlight that the induction of a stable pluripotent state, and the deriving cell instability, severely limits their use in regenerative medicine.

The new proposed method of epigenetic cell conversion demonstrated that it is possible to dynamically interact with cell genotype and phenotype through the use of epigenetic modifiers. This approach allows to directly convert a terminally differentiated cells into a different cell type, without the use of transgenes, and increase cell plasticity only for a short and transient period, and avoid the induction of a stable pluripotent state. This makes epigenetic conversion a very promising tool for regenerative medicine. Furthermore, the results obtained indicate that this protocol is robust since it was successfully applied to different cell types as well as in several species [3–5].

All this evidences support for the importance of epigenetic related approaches widen their application both to human as well as to veterinary regenerative medicine for the cure of several and diverse degenerative diseases.

## Abbreviations

5-aza-CR: 5-Azacytidine
ciNPC: Chemical-induced neural progenitor cells
DNMTs: DNA methyltransferases
DUBs: Deubiquitinases
EB: Embryoid bodies
ESC: Embryonic stem cell
GM-CSF: Granulocyte-macrophage colony-stimulating factor
HATs: Histone acetyltransferases
HDACs: Histone deacetylases
iPSCs: Induced pluripotent cell
MSC: Mesenchymal stromal cells
PKMTs: Lysine methyltransferases
PRMTs: Arginine methyltransferases
SCF: Stem cell factor
SCID: Immunodeficient severe combined immunodeficiency
TCP: Tranylcypromine
VEGF: Vascular endothelial growth factor
VPA: Valproic acid
